# A Network-Based Approach to Prioritize Results from Genome-Wide Association Studies

**DOI:** 10.1371/journal.pone.0024220

**Published:** 2011-09-06

**Authors:** Nirmala Akula, Ancha Baranova, Donald Seto, Jeffrey Solka, Michael A. Nalls, Andrew Singleton, Luigi Ferrucci, Toshiko Tanaka, Stefania Bandinelli, Yoon Shin Cho, Young Jin Kim, Jong-Young Lee, Bok-Ghee Han, Bipolar Disorder Genome Study (BiGS) Consortium, The Wellcome Trust Case-Control Consortium, Francis J. McMahon

**Affiliations:** 1 Mood and Anxiety Section, Human Genetics Branch, National Institute of Mental Health, National Institutes of Health, Department of Health and Human Services, Bethesda, Maryland, United States of America; 2 School of Systems Biology, College of Science, George Mason University, Fairfax, Virginia, United States of America; 3 Research Center for Medical Genetics, RAMS, Moscow, Russian Federation; 4 Molecular Genetics Section, Laboratory of Neurogenetics, Intramural Research Program, National Institute on Aging, Bethesda, Maryland, United States of America; 5 Longitudinal Studies Section, Clinical Research Branch, Intramural Research Program, National Institute on Aging, National Institutes of Health, Baltimore, Maryland, United States of America; 6 Geriatric Unit, Azienda Sanitaria di Firenze, Florence, Italy; 7 Center for Genome Science, National Institute of Health, Seoul, Korea; Aarhus University, Denmark

## Abstract

Genome-wide association studies (GWAS) are a valuable approach to understanding the genetic basis of complex traits. One of the challenges of GWAS is the translation of genetic association results into biological hypotheses suitable for further investigation in the laboratory. To address this challenge, we introduce Network Interface Miner for Multigenic Interactions (NIMMI), a network-based method that combines GWAS data with human protein-protein interaction data (PPI). NIMMI builds biological networks weighted by connectivity, which is estimated by use of a modification of the Google PageRank algorithm. These weights are then combined with genetic association p-values derived from GWAS, producing what we call ‘trait prioritized sub-networks.’ As a proof of principle, NIMMI was tested on three GWAS datasets previously analyzed for height, a classical polygenic trait. Despite differences in sample size and ancestry, NIMMI captured 95% of the known height associated genes within the top 20% of ranked sub-networks, far better than what could be achieved by a single-locus approach. The top 2% of NIMMI height-prioritized sub-networks were significantly enriched for genes involved in transcription, signal transduction, transport, and gene expression, as well as nucleic acid, phosphate, protein, and zinc metabolism. All of these sub-networks were ranked near the top across all three height GWAS datasets we tested. We also tested NIMMI on a categorical phenotype, Crohn’s disease. NIMMI prioritized sub-networks involved in B- and T-cell receptor, chemokine, interleukin, and other pathways consistent with the known autoimmune nature of Crohn’s disease. NIMMI is a simple, user-friendly, open-source software tool that efficiently combines genetic association data with biological networks, translating GWAS findings into biological hypotheses.

## Introduction

Genome-wide association studies (GWAS) have greatly facilitated the identification of genes involved in complex phenotypes [1], [2]. However, replication of association findings has often been difficult, probably reflecting the relatively small effects of individual markers, and the genetic heterogeneity of complex traits [3]. The critical challenge now is to understand how multiple, modestly-associated genes interact to influence a phenotype [4]–[6]. Many studies have shown that there is a strong relationship between gene function and phenotype, and that functionally-related genes are more likely to interact [7]–[18]. Inspired by this insight, we undertook a systems-biology approach to identify and prioritize groups of functionally-related genes that are enriched for genetic variants associated with a trait, what we call ‘trait prioritized sub-networks.’

Previously described network and pathway-based methods of GWAS data are useful, but have limitations. Most 1) use licensed software, which is often costly and lacks transparency [19]–[22]; 2) depend on publicly available pathway databases that rely on a limited number of available pathways (<500) and that often ignore protein-protein interactions (PPIs) for recently studied genes [23]–[30]; 3) rely on simulated or model organism data only [11], [31]; 4) require knowledge of programming [32]; or 5) limit the number of input genes [33]. Since signals with small effects not detectable at conventional levels of significance may account for substantial heritability [34], methods that can include all signals without arbitrary thresholds of statistical significance are needed. Such methods should extract more information from GWAS data by identifying susceptibility genes that have functional similarity. We hypothesized that such an approach might lead to a higher rate of replication in independent datasets, compared to studies that rely only on single markers. Replicated findings are more likely to generate sound biological hypotheses for subsequent laboratory studies.

To this end, we developed a novel software tool called Network Interface Miner for Multigenic Interactions (NIMMI). This tool generates biological networks using human PPI data, where proteins are considered as nodes and the interactions between proteins as edges. NIMMI assumes that proteins that show more interactions with other proteins in the same network (i.e., have higher connectivity) are more important than proteins with fewer interactions, and weights each protein by use of a modification of the Google PageRank algorithm [35]. This algorithm ranks proteins in much the same way as the popular search engine ranks websites on the internet, giving greater weight to proteins with more connections to other proteins, especially those that are themselves highly linked to additional proteins. Unlike the original Google PageRank algorithm, this modified algorithm uses the PPI data to calculate a “damping factor” dynamically for every gene in a network, accounting for differences in the topology of biological networks compared to computerized networks. To our knowledge, this approach has never been tested on biological networks. NIMMI combines these weights with the association signals from a GWAS to identify trait prioritized sub-networks. In this study we tested NIMMI in three GWAS datasets analyzed to assess genetic contributions to height, a classical polygenic trait. We further validate the method in a categorical phenotype, Crohn’s disease. The results demonstrate that NIMMI can effectively identify genes involved in quantitative and categorical traits and group them into biologically-plausible networks that are highly replicable across independent studies.

## Results

### Summary of the statistical approach

NIMMI is a network-based approach that relies on three basic assumptions: 1) Genes, rather than SNPs are the functional units in biology; 2) Genes do not work in isolation, thus genes whose protein products show more interactions with other proteins in the same network (i.e., higher connectivity) are more important than proteins with fewer interactions; and 3) genetic association results for a trait and protein interactions within a network are complementary forms of information, reflecting a role for that network in that trait [36]–[38].

**Figure 1:**
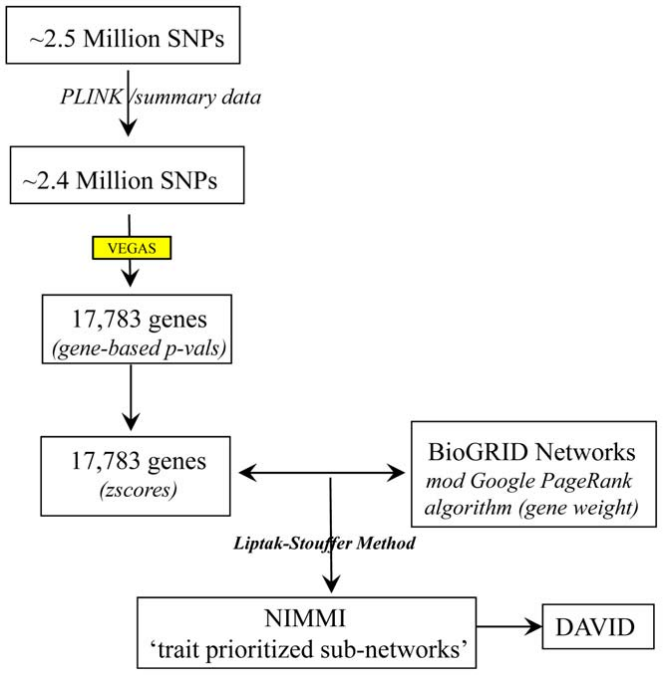
NIMMI flowchart. An overview of the dataflow in NIMMI is shown in Figure 1. The data shown here is drawn from the InCHIANTI height GWAS dataset. Approximately 2.5 million SNPs were analyzed using PLINK setting the parameters as specified under GWAS data module (see Design and Implementation). This resulted in ∼2.4 million SNPs with association p-values, which were then assigned to 17,783 genes. Gene assignment and gene-based p-values were calculated using VEGAS. These gene-based p-values were converted to z-scores and combined with gene weights (calculated by the modified Google PageRank algorithm) in the network using the Liptak-Stouffer method to identify the ‘trait prioritized sub-networks’ that were evaluated in DAVID.

NIMMI prioritizes biological networks in three key steps. First networks are identified by use of human interactome data. Proteins are represented as nodes and interactions are represented as edges. Here we assumed that each gene corresponds to a single protein and used human protein-protein interaction (PPI) data to build the networks, but in principle any data that relates one gene to another could be used. Each gene in the same network is assigned a weight (w_i_) based on connectivity to other genes in the same network, using a modification of the Google PageRank algorithm. Second, gene-based association p-values are calculated. Here we applied the Versatile Gene-based association study tool (VEGAS) (http://gump.qimr.edu.au/VEGAS/) [39] to GWAS data, but any method for mapping a SNP to a gene could be used. The gene-based p-value was converted to a z-score (z_i_) and combined with w_i_ to generate the network-weighted score for that gene. We used the Liptak-Stouffer method, which allowed us to weight the association p-value by the square-root of the sample size. Third, high-scoring genes are combined into what we call ‘trait prioritized sub-networks’, which were further tested by DAVID (http://david.abcc.ncifcrf.gov/) [40], [41], a publicly available bioinformatics tool that identifies functionally related groups of genes. A flowchart of NIMMI’s analysis steps is shown in Figure 1.

### Comparison of single-locus method with NIMMI systems approach

In order to compare ranking by single-locus analysis with NIMMI, three independent height GWAS datasets were analyzed using both single-locus ranking and NIMMI network ranking methods. Percentile ranks of 34 candidate genes for height that were deemed confirmed candidates in recent review of GWAS for height were used as a standard of comparison [42]–[44].

**Figure 2:**
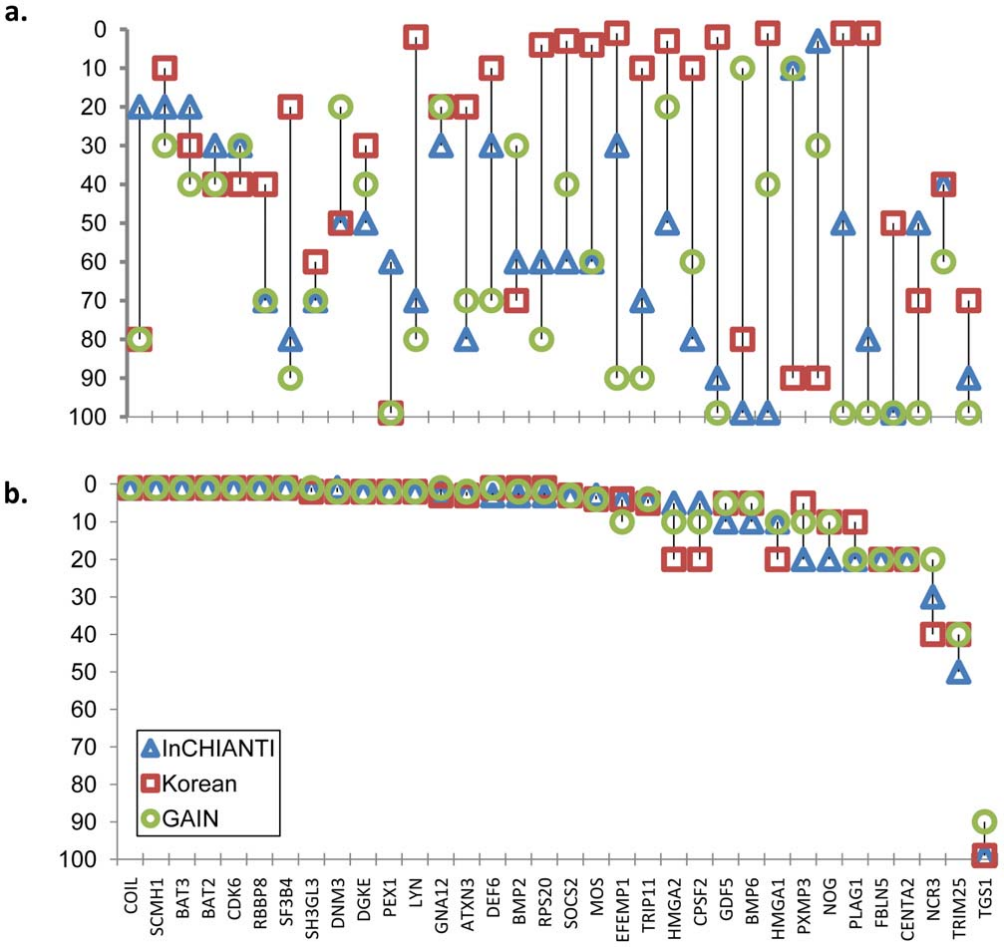
Comparison of gene-based percentile ranks with NIMMI’s network percentile ranks. The x-axis shows the candidate genes for height and the y-axis shows the percentile rank. Blue triangles represent the InCHIANTI GWAS dataset, red squares represent the Korean height GWAS dataset and green circles represent the GAIN Controls height GWAS dataset. Figure 2a shows the single-locus ranking and Figure 2b shows NIMMI network-based ranking for 34 height candidate genes.

The relative ranking of genes based on gene-wise association p-values alone was highly sample dependent, and ranks varied substantially in each GWAS dataset (Figure 2a). For the three height GWAS datasets we present the association p-values of susceptibility genes, gene-wise ranking, gene-wise percentile ranks (gene-wise PR), NIMMI-network ranking and NIMMI percentile ranks (Network PR) in Table S1. In contrast, the NIMMI-network ranking was very stable for 95% of the genes, despite differences in sample size and ancestry among the three height datasets (Figure 2b). Most of the confirmed height-associated candidate genes consistently fell in the top 2^nd^–5^th^ percentile of the NIMMI ranking, and 95% of the genes fell in the top 20^th^ percentile of all three datasets. For example, SCMH1 and CDK6, which belong to the same PPI network, were consistently ranked in the 1^st^ percentile in all three datasets. This demonstrates that NIMMI’s gene ranks are highly replicable and more stable across populations than those based on gene-based association p-values alone.

### Identification and prioritization of ‘trait prioritized sub-networks’ for height GWAS datasets

**Figure 3:**
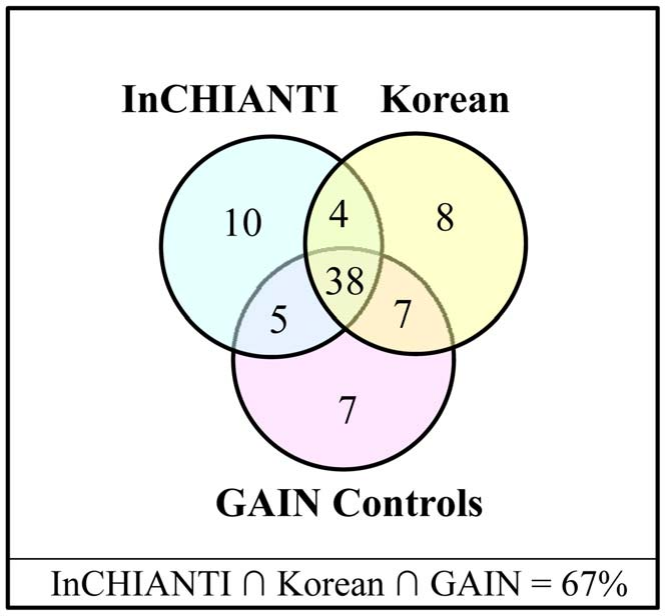
Network overlap. Top 2% overlap of NIMMI prioritized networks in InCHIANTI, Korean and GAIN controls datasets shows 38 networks that are common to all three datasets. Five networks are common to InCHIANTI and GAIN controls datasets only. Korean and GAIN controls datasets have seven networks in common and four networks are common between InCHIANTI and Korean datasets. Ten networks are specific to InCHIANTI dataset, whereas Korean and GAIN controls datasets have 8 and 7 networks, respectively.

Since 50% of the confirmed height-associated candidate genes (shown in Figure 2b) consistently fall in the top 2% of the NIMMI-ranked networks, these networks were compared in the three height datasets. A total of 38 ‘height prioritized sub-networks’ were generated, which consistently replicated across the three datasets (Figure 3). There were 7 to 10 sub-networks that appeared to be specific to each dataset and 4 to 7 sub-networks that were common to any two datasets. The 38 height prioritized sub-networks common to all three datasets were further evaluated for gene-set enrichment using DAVID.

For each NIMMI prioritized sub-network, the p-values (corrected for the total number of genes in a GWAS and for the total number of networks in each of the datasets) are presented in Table S2. A maximum of two significant Gene Ontology (GO) terms generated by DAVID are shown, along with the specific GO term, the number of genes associated with that GO term, and the corrected gene-set enrichment p-value (see Methods for GO term selection criteria). For example, one sub-network includes a total of 129 genes, of which 76 genes are involved in gene expression and 56 are involved in nucleic acid metabolism. Nineteen of the 38 sub-networks prioritized by NIMMI were significantly enriched for genes involved in nucleic acid metabolism. Eight sub-networks were enriched for genes that regulate gene expression and 12 sub-networks were enriched for zinc metabolism. Other associated GO terms implicated by NIMMI were transcription, signal transduction, transport, and phosphate and protein metabolism. Four sub-networks were excluded because they were not associated with any GO terms (not shown in Table S2).

### NIMMI analysis of randomized data

Some networks identified by NIMMI may represent general relationships among well-studied genes that arise frequently due to “small-world” effects. To estimate the impact of this phenomenon in our data, we re-analyzed the height GWAS datasets after randomization by two methods: 1. Randomization of the network nodes; and 2. Permutation of gene labels in the GWAS data. Two sub-networks appeared consistently in the random networks analysis. Eight additional sub-networks appeared in >50% of NIMMI runs performed on the randomized GWAS results (Table S3). NIMMI analysis of randomized data is thus an important step in the identification of sub-networks that are most deserving of further study.

### Comparison of NIMMI prioritized height sub-networks with Cytoscape

The three height GWAS datasets were also analyzed in Cytoscape using jActive modules and BiNGO plugins (as described in Baranzini et al [33]). Table 1 shows the 9 GO terms that were prioritized by either NIMMI or Cytoscape. There was substantial agreement between the two methods, with 7 out of 9 GO terms identified by both methods.

**Table 1:** NIMMI 'height prioritized sub-networks' vs. Cytoscape.

GO-terms*	NIMMI sub-networks	Cytoscape sub-networks
E	x	x
H		x
M	x	x
N	x	x
P	x	x
R	x	x
S	x	x
T	x	x
Z	x	

* E-Gene Expression; H - Steroid Hormone receptor signaling; M-Protein metabolic process/protein modification process; N-Nucleic acid metabolism/Nucliec acid binding/DNA-Replication; P-Phosphate/phosphorus metabolic process; R-RNA processing/RNA binding/RNA metabolic process/RNA splicing/Transcription/Transcription Regulation; S-Signal transduction/Intracellular signaling/Cell communication; T-Transport/localization; Z-metal ion binding/zinc ion binding.

### Identification and prioritization of Crohn’s disease sub-networks by NIMMI

To test the performance of NIMMI with a categorical trait, we analyzed a published GWAS based on a case-control sample studied for Crohn’s disease, an autoimmune disorder that has yielded about 20 risk loci by GWAS. NIMMI prioritized nine sub-networks that were significantly enriched for the GO terms “apoptosis”, “response to organic substance”, “intracellular signaling”, “gene expression”, “nucleic acid metabolism”, “RNA metabolism”, and “protein metabolism” (Table 2). KEGG and BioCarta pathway analysis of these nine prioritized sub-networks showed significant enrichment of apoptosis, B-cell receptor, T-cell receptor, chemokine, IL-2, IL-6, Jak-STAT, Wnt and TPO signaling pathways. These results are consistent with the known autoimmune nature of Crohn’s disease. A complete list of significantly enriched pathways is presented in Table 3.

**Table 2:** NIMMI prioritized sub-networks for Crohn's Disease.

Crohn's Disease Bonf. corrPval	David GO Set 1*	Genes in Set1	Enrichment Pval	David GO Set 2*	Genes in Set2	Enrichment Pval	David GO Set 3*	Genes in Set3	Enrichment Pval
9.51E-14	A	29/96	3.50E-08	N	31/96	7.30E-05	M	26/96	1.90E-10
2.12E-13		27/107	1.90E-03		33/107	1.70E-03	R	27/107	4.40E-11
3.30E-13		28/110	2.60E-03	O	27/110	1.00E-06	S	38/110	1.50E-07
3.53E-13		30/91	7.30E-07		23/91	4.90E-04			
3.23E-16	R	65/115	1.90E-20	O	28/115	4.90E-04	E	35/115	3.80E-15
4.10E-13		74/152	2.50E-18	N	41/152	9.00E-15	S	38/152	1.50E-04
2.89E-13	N	31/89	9.40E-05						
2.08E-14		31/123	1.80E-02						

A-Apoptosis; E-Gene Expression; M-Protein metabolic process/protein modification process; N-Nucleic acid metabolism/Nucliec acid binding/DNA-Replication; O-response to organic substance; R-RNA processing/RNA binding/RNA metabolic process/RNA splicing/Transcription/Transcription Regulation; S-Signal transduction/Intracellular signaling/Cell communication.

**Table 3:** NIMMI prioritized Crohn's Disease enriched KEGG/BioCarta pathways.

enriched KEGG/BioCarta pathways	enrichment p-value
Adherens junction	8.70E-07
Apoptosis	3.50E-05
B-cell receptor signaling	1.10E-02
Cell cycle	1.70E-07
Chemokine signaling	9.90E-03
Control of gene expression by vitamin D receptor	1.30E-07
EGF signaling	7.60E-04
ErbB signaling	8.10E-06
Erk1/Erk2 Mapk signaling	9.20E-05
Fc gamma R-mediated phagocytosis	5.80E-14
Focal adhesion	3.20E-06
IL-2 Receptor Beta Chain in T cell Activation	1.60E-02
IL6 signaling	2.80E-03
Insulin signaling	2.90E-04
Jak-STAT signaling	2.70E-03
Neurotrophin signaling	1.20E-02
p53 signaling	1.20E-03
Pathways in cancer	6.30E-11
Pelp1 Modulation of Estrogen Receptor Activity	1.20E-04
Role of PPAR-gamma Coactivators in Obesity and Thermogenesis	2.40E-04
T Cytotoxic Cell Surface Molecules	8.00E-03
T-cell receptor signaling	1.50E-04
TPO signaling	7.30E-03
Wnt signaling	1.70E-03

## Discussion

NIMMI is a simple and efficient software tool that allows researchers to prioritize their GWAS results based on the functional relationships of the associated genes. To our knowledge, NIMMI is the first software tool that maps all the genes in a GWAS dataset to human interactome data using a modified Google PageRank algorithm. With NIMMI it is possible to identify ‘trait prioritized sub-networks’ in complex, multigenic traits and thus provide biological hypotheses for further study.

We hypothesized that NIMMI would produce more robust findings than single-locus analyses. To test this hypothesis, NIMMI was run on three independent samples rated for the classic polygenic trait of height. NIMMI produced a list of genes with very consistent ranking across all 3 datasets. This level of reproducibility was not achieved with gene-based analysis, probably reflecting small effect sizes of individual loci and differing sample sizes, reducing power to detect true signals. Despite population and sample size differences, NIMMI also identified networks that were enriched with confirmed height associated candidate genes. Furthermore, when height associated candidate genes were analyzed in DAVID, there were no significantly enriched GO terms suggesting that the functional relationships between genes that NIMMI networks represent could not be identified with single-locus analyses alone.

NIMMI’s approach is unique. Previous studies that have used the Google PageRank algorithm to rank genes in a network relied on fixed damping factor values (0.5≤*d*≤0.95) (see Methods) [45], [46], [47]. Research by Fu et al has shown that a fixed damping factor can result in inconsistent ranking of the nodes in a network. Given that a flexible damping factor is needed, one of the natural approaches is to calculate it dynamically by using the ratio of interactions between neighboring genes [35]. Hence, NIMMI calculates the damping factor dynamically for every gene in a network which may be more appropriate for biological networks than the fixed damping factor typically used for ranking pages on the internet.

Most of the literature published on network and pathway-based approaches has focused on statistically significant findings from GWAS studies for replication or for downstream analysis [20], [21], [25], [27], [28], [33], [48]. These studies are limited by the problem of finding an optimal p-value threshold. If the p-value threshold is set too low, then the number of genes might be too few to create a biological network and to find associated pathways [49]. Sets of findings with higher p-value thresholds, on the other hand, will contain more false positives. The magnitude of this problem was illustrated by the results of the International Schizophrenia GWAS Consortium, where optimal discrimination between cases and controls was achieved only after the inclusion of over 70,000 markers with p-values as high as 0.2 [34]. A major advantage of NIMMI’s approach is that it includes all the findings in a GWAS dataset, weighting the findings by p-value and other factors that users may specify (such as effect size). This reduces the “top hits” selection bias. An example may illustrate this point. The gene BMP2, which encodes bone morphogenetic protein 2, has been implicated in several height GWAS studies [42], but it is not significant in any of the GWAS datasets used in the current study (gene-wise p-values of 0.57, 0.61 and 0.27). Although BMP2 plays a major role in bone development, this gene would not have been selected for downstream analysis with the classical p-value approach. However, NIMMI’s network ranking correctly places this gene in the top 1^st^–3^rd^ percentile. This is because NIMMI takes advantage of signals in genes whose protein products interact with BMP2, making NIMMI sensitive to statistical significance in any individual dataset.

The goal of NIMMI is to prioritize networks for further study. To aid decisions as to which networks to pursue, NIMMI can be applied to randomized networks and GWAS data. Of the 38 ‘height prioritized sub-networks’ that successfully replicated in the three GWAS datasets, 2 appeared consistently in the random networks analysis, suggested that the Type I error rate was about 5% at the network level. When NIMMI was used to analyze 10,000 randomized GWAS datasets, a number of sub-networks appeared in >50% of the results sets (Table S3). We suggest that the users assess their own results in this way and ignore sub-networks that occur frequently in the results derived from randomized data. It is likely that such sub-networks represent general relationships among well-studied genes that arise frequently due to “small-world” effects. Sub-networks that occur rarely in the results derived from randomized data appear to have good trait specificity. For example, 16 height-prioritized sub-networks that were observed in <5% of the randomized results included 7 sub-networks that were significantly enriched for zinc metabolism. This GO term was enriched only for height and not Crohn’s disease. Zinc plays an important role in human growth. Studies in rats and tissue culture confirm that zinc stimulates DNA synthesis and protein synthesis in bone development [50], [51]. Furthermore, zinc is a co-factor for zinc finger proteins that bind to methylated DNA to suppress transcription, thus regulating gene expression and protein synthesis [52].

Other GO terms significantly enriched among the height-prioritized sub-networks include nucleic acid metabolism, transcription, gene expression, signal transduction and transport, proteosome and protein metabolism. The effects of growth hormone on protein metabolism are well-documented in the literature, in both human and animal models. These studies suggest that growth hormone stimulates protein synthesis and decreases protein catabolism, a process that mainly occurs in the proteosome [53]–[56]. Additionally, keywords such as gene expression, signaling and proteases have recently been associated with height markers by GRAIL [57], a statistical text mining approach. Although most of the NIMMI detected ‘height prioritized sub-networks’ are well-supported by the literature, further functional studies are required to confirm these results.

NIMMI is reasonably sensitive in detecting true trait-related genes. For example, Table S1 includes 34 height associated genes, 16 of which were among the 1,232 genes identified within the top 2% of height prioritized sub-networks by NIMMI. This is a highly significant overlap, given that the total number of genes in all sub-networks created by NIMMI is 6035 (p-value = 1.11×10^−4^, hypergeometric test). Although 38 sub-networks were prioritized for height, there is substantial overlap among these sub-networks (75%-90%). Each functional category consists of ≤100 genes. There is also overlap of genes between functional categories. For example, the category “gene expression” (E) includes ∼100 genes, and “transcription” (R) includes ∼75 genes, but at least 50% of the genes in set E overlap with those in set R.

Some of the functional categories (nucleic acid metabolism, gene expression) overlap between height and Crohn’s disease prioritized sub-networks. These are broad categories and may reflect a true pathway overlap for these two otherwise disparate phenotypes [58]. A few functional categores, such as zinc metabolism and transport, were specific to height. Several functional categories were specific to Crohn’s disease, including apoptosis, response to organic substance, Jak-Stat signalling and autoimmune pathways (B-cell receptor, T-cell receptor signaling,etc), consistent with the auto-immune nature of this disease. Although these pathways were detected by other studies [59], additional research is necessary to confirm the NIMMI results in additional samples.

Comparison of NIMMI prioritized height sub-networks to those from Cytoscape jActive modules illustrate that NIMMI gave similar results to the greedy algorithm implemented in Cytoscape. A major limitation of Cytoscape jActive modules is that it requires the input gene set be limited to those with p-value ≤0.05. NIMMI considers all genes implicated in a GWAS dataset, with no p-value threshold required. Consideration of all genes in the network analysis is important because GWAS p-values are inconsistent, especially when sample sizes are small. Including all genes in the Cytoscape analysis resulted in more than 1500 significantly associated GO terms, some of which could be false positives. Furthermore, NIMMI prioritizes trait networks within 3 seconds, which is 10 times faster than what we could achieve with Cytoscape. Cytoscape jActive modules and BiNGO also need more user intervention and formatting than is ordinarily necessary for NIMMI.

The goal of GWAS is to help illuminate the underlying molecular mechanism of a particular phenotype. To maximize the information provided by a GWAS, it is important to integrate functional data with GWAS results. NIMMI is a user-friendly software tool that will help researchers in their post-GWAS decisions by prioritizing genes and networks that are of the highest biological relevance.

### Limitations

Some important limitations of network and pathway-based approaches should be mentioned. SNPs do not always bear a clear relationship to a particular gene, and may occur in non-genic regions. Some widely studied genes appear to have relatively greater connectivity, while less studied genes have less, due largely to publication bias. PPIs are sometimes inconsistent, or tissue dependent. Functional annotation systems, such as GO, have not yet been experimentally confirmed for most genes. In spite of these limitations, network and pathway-based approaches provide a unique insight into biology that is often not immediately evident in the GWAS results. As additional genome-wide functional studies are completed in the field, the quality of network and pathway information is likely to improve [29], [60]–[63].

### Conclusions

NIMMI is an open-source tool that takes into account information on biological relationships to help interpret GWAS data and to prioritize trait networks for further study. NIMMI offers several advantages over other network and pathway-based approaches. The results of this study demonstrate that NIMMI can identify important genes involved in a multi-genic trait with a high degree of consistency and reproducibility, even across datasets of differing size and ancestry.

The main aim of NIMMI is to help investigators prioritize genes and networks related to a particular phenotype after a GWAS. Although there are limitations to this approach, protein-protein networks and pathways are an excellent source of biological information that, when combined with genomics, could lead to a better understanding of molecular mechanisms. NIMMI efficiently combines genetic association data with protein networks, thus helping to effectively translate GWAS findings into biological hypotheses.

## Materials and Methods

### Height GWAS datasets

A total of three independent GWAS height samples were analyzed by NIMMI.

#### The Invechhiare in Chianti (InCHIANTI) GWAS dataset

The InCHIANTI Study is a population-based epidemiological cohort study in the Chianti region of Tuscany, Italy. The study employs two clinical sites, in the towns of Greve and Bagno a Ripoli, with participants recruited from the population registries of these immediate areas. Further details on this cohort have been previously published elsewhere [64]. DNA extracted from InCHIANTI participants was genotyped at the Laboratory of Neurogenetics, National Institute on Aging, using Illumina 550K beadchips. After standard QC measures, missing genotypes were imputed using MACH 1.0 software (http://www.sph.umich.edu/csg/abecasis/mach/) [65]. Maximum likelihood genotype dosages were filtered for quality of imputation prior to analysis (R^2^ from MACH>0.30). Analyses were conducted on 975 unrelated members of the InCHIANTI cohort who had height data within +/-3SD from the mean of the sample. After outliers were removed height was log transformed. PLINK (http://pngu.mgh.harvard.edu/~purcell/plink/), a whole genome association analysis toolset [66], was used for association analysis using study site and sex as covariates, resulting in a ∼2.4 million SNPs after pruning. Genomic control inflation factor (λ) was 1.008. A λ close to 1 indicates that association of markers to the phenotype is real rather than due to population stratification. A total of 17,783 genes with gene-wise p-values were used in the downstream analysis (Table 4).

**Table 4:** Summary of GWAS datasets.

Height Datasets	n	Observed [O] Imputed [I]	SNPs after QC		Total Genes
InCHIANTI	975	I	2,453,309		17,783
Korean	8,842	O	352,228		17,408
GAIN Controls	768	O	722,742		17,720

Crohn's Disease	Cases	Controls	Observed [O] Imputed [I]	SNPs after QC	Total Genes
WTCCC Crohn's	1,748	2,953	O	409,541	17,114

#### Korean GWAS dataset

Height GWAS data published by Cho et al. were obtained from the researchers [67]. Unimputed summary data for 8,842 Korean individuals was provided as well. Age, sex and study site were used as co-variates in the PLINK association analysis. λ was 1.061 and ∼350K SNPs were left after pruning, resulting in 17,408 genes being analyzed by NIMMI (Table 4).

#### Genetic Association Information Network (GAIN) Controls GWAS height dataset

GWAS data for GAIN controls was obtained from GAIN (http://www.genome.gov/19518664) under a data access agreement. Self-reported height was available for 768 individuals. Only sex was used as a co-variate and ∼720 K SNPs were left after pruning by PLINK. λ was 1.059 and a total of 17,720 genes were used in the final analysis by NIMMI (Table 4).

### Crohn’s disease (CD) GWAS dataset

Summary SNP association results for the CD GWAS dataset (∼2000 cases and ∼3000 controls) was obtained from the Wellcome Trust Case Control Consortium (WTCCC). Association analysis results are detailed elsewhere [68]. Approximately 409 K SNPs with their association p-values were assigned to 17,114 genes that were included in NIMMI network analysis (Table 4).

### Algorithm

#### Modified Google PageRank Algorithm

Each protein in a network was considered a node, and the interactions between proteins were considered edges, resulting in an undirected graph. Some of the centrality measures available to rank networks are: 1) Degree centrality, which simply counts the number of interactions to a node; 2) Betweenness centrality, where nodes which fall in the shortest path of other nodes have high betweenness; 3) Closeness centrality, which is related to the topology of the nodes in a network; and 4) Eigenvector centrality which ranks the nodes in a network based on the its interacting neighbors, i.e., it takes into account the quantity and quality of connections to a particular node [69], [70]. For example, when ranking a webpage (w_p_), Google’s PageRank algorithm not only considers the number of connections a w_p_ has, but also the connectivity of the pages linked to w_p_. Therefore, w_p_ will receive a higher rank if it is connected to other highly ranked webpages. Since proteins in a biological network should also be ranked based on the importance of their interacting partners, scoring them by eigenvector centrality seems to be a reasonable approach.

The PageRank algorithm, which is a based on eigenvector centrality, starts by creating an adjacency matrix (A) for all the proteins (nodes) in a network based on their interactions. A_i,j_ = A_j,i_ = 1 if protein ‘i’ interacts with protein ‘j and vice versa, else A_i,j_ = A_j,i_ = 0. According to the Perron-Frobenius theorem in linear algebra, for a real square matrix with positive values there exists a largest positive eigenvalue and a corresponding positive dominant eigenvector [69]. The algorithm finds the dominant eigenvector for this adjacency matrix via power iteration (Figure S1) [45], [71]–[73]. Thus, the resulting dominant eigenvector values are considered weights of proteins in a network.

The original Google PageRank algorithm suggested by Lawrance Page and Sergey Brin had a damping factor of 0.85 and excluded dangling links (nodes that have only incoming links but no outgoing links), before ranking the web networks. A damping factor is essential because it improves the speed of the algorithm and keeps it from hitting dead ends [74]–[77] (http://www.miislita.com/information-retrieval-tutorial/matrix-tutorial-3-eigenvalues-eigenvectors.html; http://www.webworkshop.net/PageRank.html). Investigators who previously applied the Google PageRank algorithm to rank SNPs or genes used a damping factor between 0.5≤d≤0.95 [45], [46]. The damping factor of 0.85 has been well evaluated for web networks and not for biological networks. Additionally, Fu et al have shown that a fixed damping factor value will lead to inconsistent ranking of nodes in a network [35]. Since all biological networks are not the same, having a fixed damping factor value for all networks might not be ideal. Furthermore, dangling links should be included while ranking a biological network. Given these limitations of the original Google PageRank algorithm, we used the modified Google PageRank algorithm suggested by Fu et al in 2006 [35] to rank the proteins in each of our 2,849 networks (see Design and Implementation).

Equation (1) depicts the modified Google PageRank algorithm
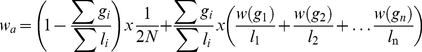
(1) where,


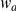
 = weight/PageRank of protein (gene) A in a network


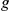
 = all proteins that interact with protein A


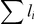
 = sum of all proteins that interact with ‘g’ proteins


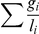
 = damping factor, which is the ratio of total proteins ‘g’ that interact with protein A to the sum of all proteins that interact with ‘g’ proteins


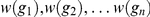
 = weight/PageRank of proteins H_1_ to H_n_ that interact with protein A


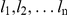
 = proteins that interact with proteins g_1_, g_2_,…… g_n_ respectively


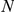
 = Total number of proteins in the network

The advantage of using the modified formula is that it considers all nodes in the network without any exclusion, and that the damping factor is calculated dynamically for every node in a network based on its interactions with neighboring nodes, making it more optimal to rank biological networks. Such an approach results in a damping factor value that is better reflective of the network. To scale the total probability of a given network between 0 and 1, the N in the above formula was doubled. Each protein in a network has a weight ranging between 0 and 1. The protein with weight closer to 1 plays an important role in the network than a protein with weight closer to 0, indicating that the higher the weight the higher the importance of a node in a network.

#### The Liptak-Stouffer method

The gene-wise association p-value, which is calculated by VEGAS, was integrated with gene weight (obtained from the modified Google PageRank algorithm) in a network. While several approaches are possible, for simplicity we chose the Liptak-Stouffer method. This method of combining p-values from independent experiments has previously been used in the analysis of genome-wide gene expression data [78]-[80] among others. At first, the association p-value of each gene was converted to its corresponding z-score as calculated in equation (2):
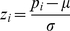
(2) where,


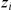
 = z-score of a particular gene


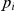
 = empirical gene-wise p-value of a particular gene in a given GWAS dataset


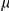
 = mean of all the p-values in a given GWAS dataset


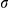
 = standard deviation of the p-values in a given GWAS dataset

Then, a combined z-score for a network was calculated using the Liptak-Stouffer formula shown in equation (3):
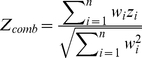
(3) where,


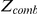
 = combined Z-score of a given network


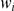
 = weight of the protein (gene) obtained from modified Google PageRank in a given network


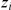
 = association z-score of the gene obtained from GWA data


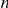
 = number of proteins (genes) in a given network

Usually, the “w_i_” in the Liptak-Stouffer formula refers to sample-size. However, we assigned gene weights based on the modified Google PageRank algorithm. A justification is provided in Methods S1 and Figure S2. The Z_comb_ is then transformed into its corresponding p-value and corrected for the number of genes in a network and total number of networks (by Bonferroni correction).

### Architecture of Network Interface Miner for Multigenic Interactions (NIMMI)

**Figure 4:**
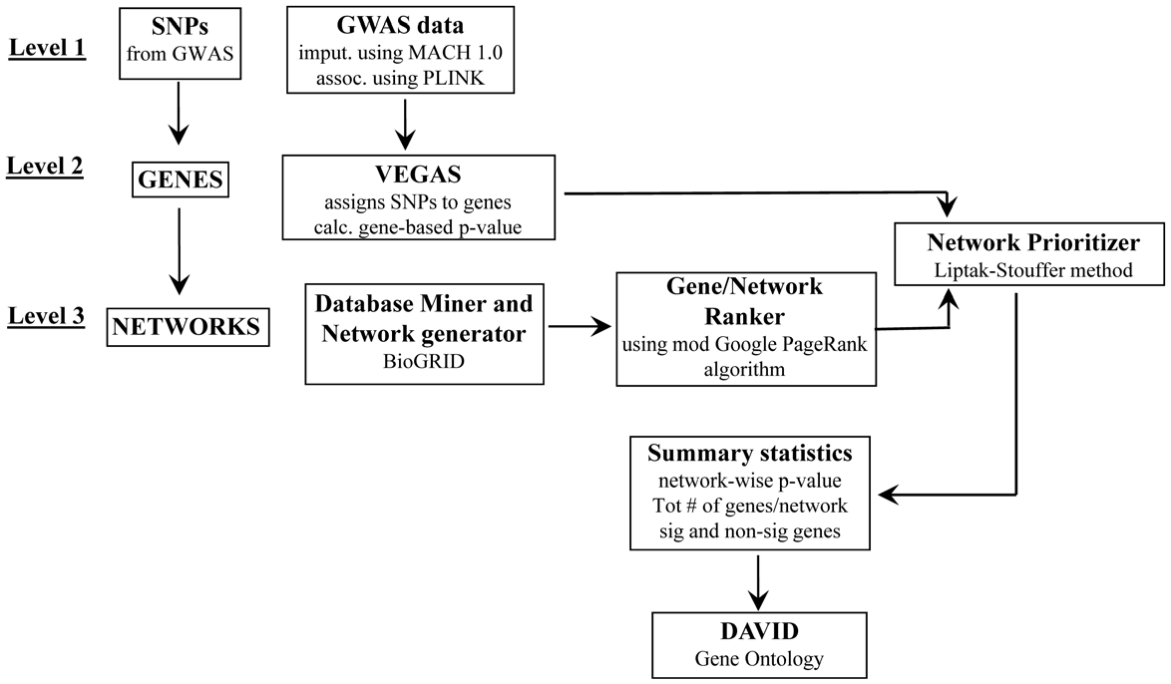
Architecture of Network Interface Miner for Multigenic Interactions (NIMMI). Network Interface Miner for Multigenic Interactions (NIMMI) consists of three levels: SNPs, Genes and Networks, and each level in turn has different modules necessary to prioritize ‘trait prioritized sub-networks’. At the SNPs level (or Level 1), the SNPs are analyzed in the GWAS data module using PLINK. The SNPs are then assigned to genes and a gene-wise p-value is calculated using VEGAS (Level 2). The Database Miner and Network generator module in Networks level (or Level 3) mine the BioGRID database for human PPIs and created two-step networks that are then ranked using the modified Google PageRank algorithm in the Gene/Network ranker and prioritizer module. The association p-value of a gene from Level 2 and gene weight from Level 3 are then combined using the Liptak-Stouffer method. The resulting ‘trait prioritized sub-networks’ are then evaluated in DAVID.

NIMMI consists of three levels: SNPs, Genes and Networks. Each level in turn has sub-modules (Figure 4). At the SNPs level (or Level 1), the SNPs were analyzed in the GWAS data module, which were then assigned to genes at GENES level (Level 2) using VEGAS, a software tool. VEGAS also calculates a gene-based p-value for each gene. The Database Miner and Network generator module in Networks level (or Level 3) mined the BioGRID database for human PPIs and created two-step networks that were then ranked using the modified Google PageRank algorithm using Gene/Network ranker module. An association gene-wise p-value of a gene from VEGAS and gene weight from Gene/Network ranker module were then combined using Liptak-Stouffer method. The resulting ‘trait prioritized sub-networks’ were then evaluated in DAVID.

#### Level 1: SNPs: GWAS data module

Individual population-based samples from the “Invechhiare in the Chianti” (InCHIANTI) study and Genetic Association Information Network (GAIN) were genotyped on Illumina 550 K and Affymetrix 6.0 microarrays respectively. Genotype information from these SNPs and phased haplotype information on a reference dataset from the HapMap Phase 2 CEU samples (http://hapmap.ncbi.nlm.nih.gov/) were used to impute the allele frequencies of SNPs that are absent on these microarrays. Imputation was performed using MACH 1.0 software. The resulting data were analyzed using PLINK. The following criteria were applied while executing PLINK:

1. SNPs with minor allele frequency (MAF)<1% were excluded.
2. SNPs that deviate from Hardy-Weinberg equilibrium (HWE) may indicate genotyping errors [81]–[83]. Hence, SNPs with HWE p-value<10^-5^ were excluded.
3. SNPs with failure rate>2% were excluded.
4. Any individuals with>2% missing genotypes were excluded.

A summary statistics file (SNP name along with its association p-value) of analyzed height GWA data was obtained from our collaborators in Korea and a similar file for Crohn’s disease GWAS dataset was obtained from WTCCC.

At the end of Level 1, GWA data consisted of a SNP name and an association p-value which are annotated to their respective genes in Level 2.

#### Level 2: GENES: Versatile Gene-based Association Study (VEGAS)

VEGAS is an open source software tool that assigns SNPs from Level 1 to their respective genes based on their position and calculates an empirical gene-based p-value using Monte Carlo simulations (≤ 1 million) (http://gump.qimr.edu.au/VEGAS/) [39]. Any SNP that falls within a 50 kb flanking region of a gene will be assigned to that particular gene. Such an assignment will capture the regulatory regions and SNPs in LD. Although this value is arbitrary, it could be modified according to user specification. When a SNP belongs to more than one gene, that particular SNP was assigned to multiple genes in that location. Given that the PPI data is incomplete, such an assignment allows us to include all the possible genes with interaction data to be included in our downstream analysis. The linkage disequilibrium for each gene is estimated using HapMap populations. For InCHIANTI and GAIN controls height GWAS dataset, and CD GWAS dataset CEU population was used as a reference dataset, whereas CHB_JPT was used as a reference for Korean height GWAS dataset.

#### Level 3: NETWORKS

This level is made up of a Database Miner and Network generator module and a Gene/Network Ranker and prioritizer module. **(1)** ***Database Miner:*** The entire human interactome consisting of 40,206 human PPIs was downloaded from the Biological General Repository for Interaction Datasets (BioGRID) database (http://www.thebiogrid.org/). After excluding human-nonhuman PPIs, a total of 38,509 human-human PPIs were left. Any PPIs detected by different experimental methods were integrated and self interacting proteins were deleted resulting in 24,101 unique human-human PPIs involving 7,646 unique proteins. These unique interactions were used to build biological networks. **(2)** ***Network generator:*** In the human PPI network, proteins are represented as nodes and the interaction between proteins are represented as edges. In order to find the optimal number of proteins per network, networks were created using single-step, two-step and three-step methods (data not shown). The median number of proteins per network with a single-step is three (too few) and with three-step was found to be 815 (too large). However, the median number of proteins per network created by two-step process was 58; this is an optimal number for computational efficiency as well as for the search space. A total of 7,646 networks were created by the network generator using the two-step method (Figure S3), of which 2,912 networks were complete subsets of other networks. Hence, these were excluded in the downstream analysis leaving 4,734 networks. These were then ranked using the modified Google PageRank algorithm (see Algorithm). Each of the 4,734 networks had between two and 1000 proteins. To reduce the search space, multiple-testing correction factor associated with the number of proteins and number of networks, and to avoid any false positive networks that could arise due to large size of the networks, we included networks that have only 20–200 proteins [29], [48], [84]. The algorithm was tested on various protein ranges in InCHIANTI dataset; this also led to the conclusion that the optimal range of proteins in a network is 20–200 (data not shown) resulting in a total of 2,849 networks. **(3)** ***Gene/Network Ranker module:*** The resulting 2,849 networks from above Network generator module were ranked using the modified Google PageRank algorithm (see Algorithm). **(4)** ***Network Prioritizer module:*** In this module the association signals were combined with gene weights using the Liptak-Stouffer method (see The Liptak-Stouffer method).

#### Summary statistics module

In this module summary statistics for each network were generated. These consisted of names of significant genes and non-significant genes, number of significant and non-significant genes; total number of genes in a network; network number; combined Z-score (Z_comb_); network p-value and corrected p-value.

#### DAVID, a functional annotation tool

To evaluate results obtained from NIMMI, genes in the top replicated networks were submitted to DAVID, a functional annotation tool [40], [41]. There were a total of 7,646 unique proteins (genes) in BioGRID. The official names of these genes were converted to Genbank accession numbers and submitted as background to DAVID. Of the 7,646 genes, DAVID found 6,327 genes and set them as background. DAVID corrects the enrichment p-values for the number of background genes submitted. Then genes belonging to a particular network were submitted and results from the functional annotation tool were selected based on the following criteria:

- Each GO category should have at least 25% gene overlap with the input list.
- Enrichment p-value for each GO term had to be significant (p≤0.05).
- Benjamini-Hochberg p-value (False discovery rate (FDR)) should be ≤0.05.
- Medium to high stringency level was selected.
- General GO terms like plasmamembrane, membrane, cytoplasm, intracellular, etc were excluded.

To keep the tables concise, the top two GO categories that fit the abovementioned criteria were presented in the results.

### Replication

Since NIMMI considers genes and networks as the functional units rather than individual SNPs, replication is expected at the gene/network level rather than at SNP level in the new sample. To test this, the networks NIMMI identified for height in the InCHIANTI GWAS dataset were tested in two independent GWAS datasets (Korean and GAIN Controls) for height.

### Randomization of networks

Networks were randomized by permutation of the node labels. Although the number of nodes and edges per network remain the same as the original network, the identities of the nodes were changed resulting in randomization of the network. By this randomization procedure hubs will not remain as hubs. We performed 100 randomizations of the original networks.

### Permuting GWAS data

In a given GWAS dataset the gene labels and the association p-values were permuted and NIMMI analysis was performed on each of the 10,000 permuted files.

### Implementation of NIMMI

The user input file can either be in PLINK format (*.ped and *.map files) or a summary file with marker names along with association p-values. VEGAS then assigns these markers to their respective genes and calculates a gene-wise association p-value. The output file from VEGAS is then input to a perl script, weightedZscore_forVegasFiles.pl, which converts the association p-values to z-scores and integrates them with gene weights in networks to obtain a combined Z-score for a network (Z_comb_), which is then converted to a p-value and corrected for the number of tests. The implementation of the modified Google PageRank algorithm in NIMMI has allowed it to identify ‘trait prioritized sub-networks’ in a given dataset within three seconds.

### Availability and Future Directions

NIMMI software along with user manual can be downloaded from http://mapgenetics.nimh.nih.gov/datashare.html.

The current version of NIMMI includes only human PPIs, but this could easily be extended to PPIs from other model organisms. Future versions will incorporate gene expression data and micro RNA studies, whenever available, which could help in further pruning of the prioritized list of networks.

## Supporting Information

Figure S1 **Power iteration.** An adjacency matrix was created based on the links of all the proteins in a network. The power iteration starts by initializing an eigenvector which is multiplied with the adjacency matrix resulting in a new eigenvector. This new eigenvector is normalized and multiplied with the original adjacency matrix until the algorithm finds a dominant eigenvector for this adjacency matrix.
(PDF)

Figure S2 **Standard error of the mean versus network size.** The x-axis shows the number of genes in a network and y-axis shows the standard error of the mean (SEM).
(PDF)

Figure S3 **Constructing Two-step networks.** Building a two-step network starts with one protein-protein interaction (protein1-protein2). In STEP1 all the proteins interacting with protein1 and protein 2 are added to the network i.e., protein 3 interacts with protein 1 and protein 4 interacts with protein 1 and protein 2, so it is linked to both these proteins. In STEP2 all proteins interacting with proteins in STEP1 are added to the network, for e.g., proteins 6, 7 and 8 interact with protein 4 and protein 5 interacts with protein 3.
(PDF)

Methods S1 **Rationale for using gene weight “**w_i_ **” in Liptak-Stouffer method.**
(DOC)

Table S1 **Comparison of single-locus ranking with NIMMI network ranking.**
(DOC)

Table S2 **‘Trait prioritized sub-networks’ for height.**
(DOC)

Table S3 **Empirical p-values of height-prioritized sub-networks.**
(DOC)
