# Supplementary figures and images for: A Network-Based Approach to Prioritize Results from Genome-Wide Association Studies
Source: PLoS One. 2011 Sep 6;6(9):e24220. doi: 10.1371/journal.pone.0024220 (PMC3168369; doi:10.1371/journal.pone.0024220)

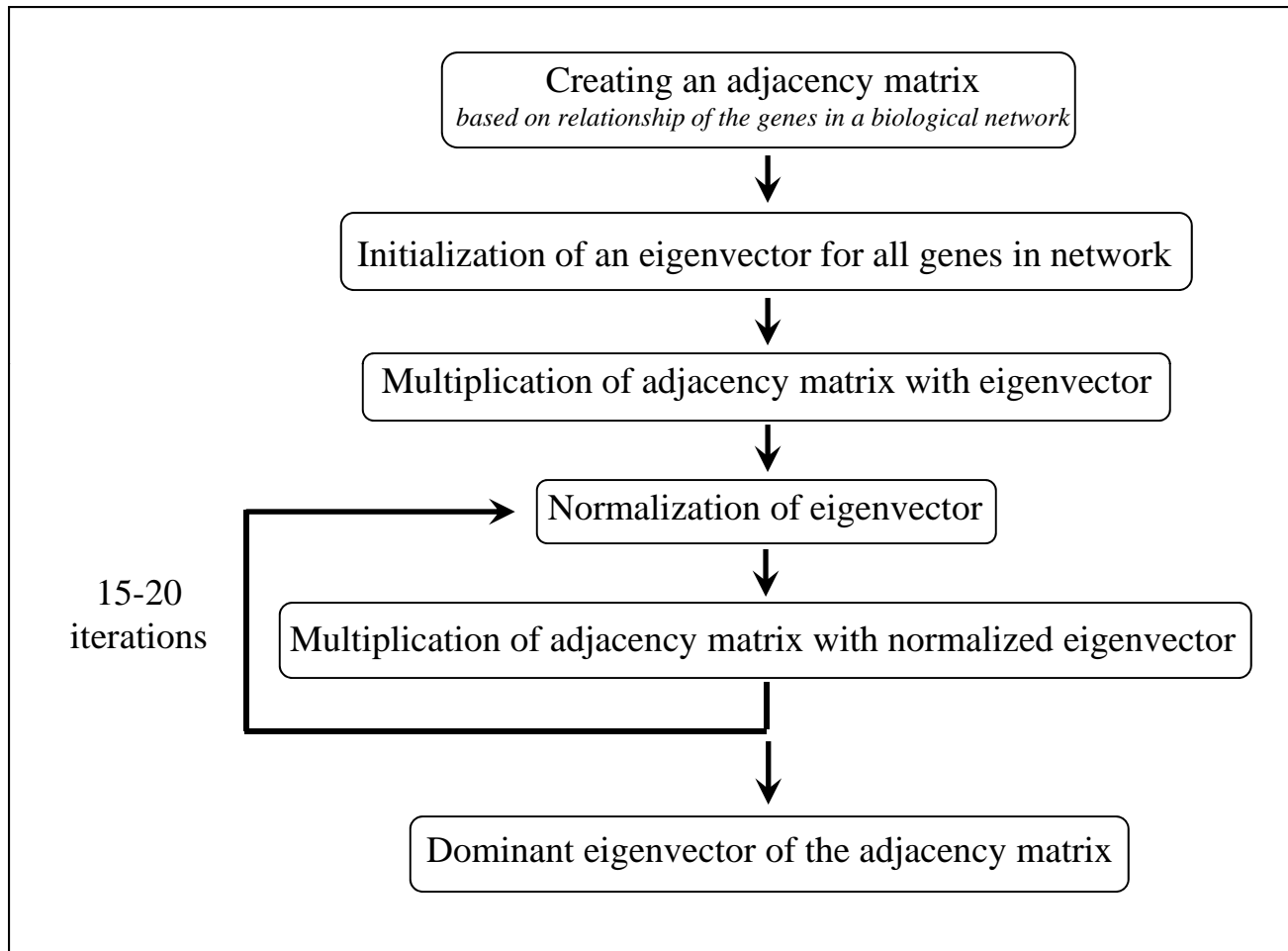

Supplement: Figure S1 — Power iteration. An adjacency matrix was created based on the links of all the proteins in a network. The power iteration starts by initializing an eigenvector which is multiplied with the adjacency matrix resulting in a new eigenvector. This new eigenvector is normalized and multiplied with the original adjacency matrix until the algorithm finds a dominant eigenvector for this adjacency matrix. (PDF) [file pone.0024220.s001.pdf]

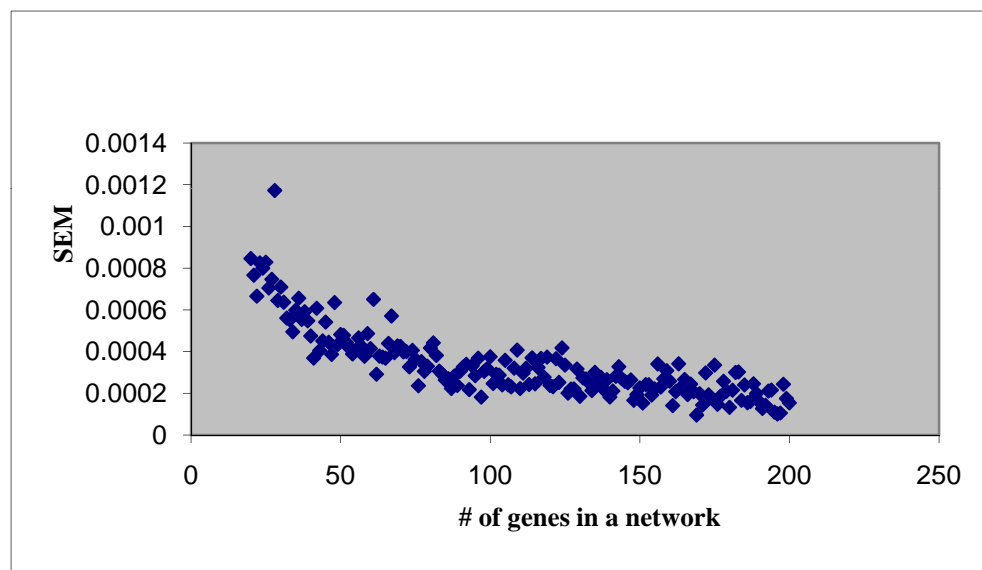

Supplement: Figure S2 — Standard error of the mean versus network size. The x-axis shows the number of genes in a network and y-axis shows the standard error of the mean (SEM). (PDF) [file pone.0024220.s002.pdf]

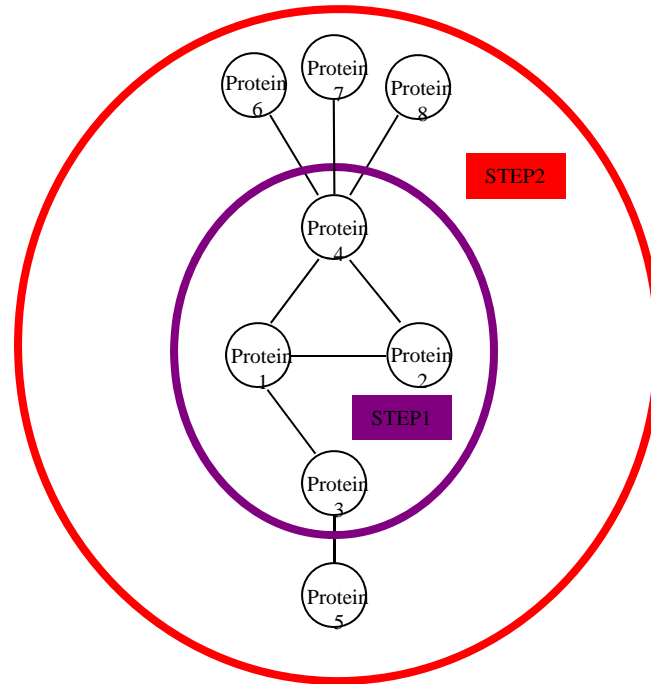

Supplement: Figure S3 — Constructing Two-step networks. Building a two-step network starts with one protein-protein interaction (protein1-protein2). In STEP1 all the proteins interacting with protein1 and protein 2 are added to the network i.e., protein 3 interacts with protein 1 and protein 4 interacts with protein 1 and protein 2, so it is linked to both these proteins. In STEP2 all proteins interacting with proteins in STEP1 are added to the network, for e.g., proteins 6, 7 and 8 interact with protein 4 and protein 5 interacts with protein 3. (PDF) [file pone.0024220.s003.pdf]
