# Supplementary material for: A Network-Based Approach to Prioritize Results from Genome-Wide Association Studies
Source: PLoS One. 2011 Sep 6;6(9):e24220. doi: 10.1371/journal.pone.0024220 (PMC3168369; doi:10.1371/journal.pone.0024220)
Supplement: Methods S1 — Rationale for using gene weight “wi ” in Liptak-Stouffer method. (DOC) [file pone.0024220.s004.doc]

**Supporting Information**

**Methods S1**

**Rationale for using gene weight “w_i_” in Liptak-Stouffer method**

Frequently, the “w_i_” in the Liptak-Stouffer formula refers to the weight of the study sample. However, if the gene weights in a network follow the properties of a sample size, then using the gene weights for “w_i_” in the Liptak-Stouffer method should be justified. The following points argue that gene weights indeed follow the properties of sample size:

1. As power to detect an associated gene increases with sample size, similarly, the power to detect a given protein in a network increases as network size increases.
2. Similar to sample size, as network size increases the standard error of the mean (SEM) of network weights decreases (Figure S2).
