# Supplementary material for: A Network-Based Approach to Prioritize Results from Genome-Wide Association Studies
Source: PLoS One. 2011 Sep 6;6(9):e24220. doi: 10.1371/journal.pone.0024220 (PMC3168369; doi:10.1371/journal.pone.0024220)
Supplement: Table S1 — Comparison of single-locus ranking with NIMMI network ranking. (DOC) [file pone.0024220.s005.doc]

**Table S1: Comparison of single-locus ranking with NIMMI network ranking**

| **Height genes** | **InCHIANTI GWAS Data (975 inds, Imputed)** | | | | | **Korean GWAS Data (~9000 inds, Unimputed)** | | | | | **GAIN Ctrls GWAS Data (~768 inds, Unimputed)** | | | | |
| --- | --- | --- | --- | --- | --- | --- | --- | --- | --- | --- | --- | --- | --- | --- | --- |
|  | **VEGAS Gene-based ranking (17,295 genes)** | | | **NIMMI ranking (2,849 networks)** | | **VEGAS Gene-based ranking (14,434 genes)** | | | **NIMMI ranking (2,849 networks)** | | **VEGAS Gene-based ranking (16,954 genes)** | | | **NIMMI ranking (2,849 networks)** | |
|  | Gene wise P-val | Gene wise Rank | Gene wise PR | Network Rank | Network PR | Gene wise P-val | Gene wise Rank | Gene wise PR | Network Rank | Network PR | Gene wise P-val | Gene wise Rank | Gene wise PR | Network Rank | Network PR |
| COIL | 0.111 | 1,853 | 20 | 5 | 1 | 0.762 | 13,853 | 80 | 5 | 1 | 0.771 | 13,567 | 80 | 1 | 1 |
| SCMH1 | 0.121 | 2,026 | 20 | 2 | 1 | 0.059 | 1,618 | 10 | 7 | 1 | 0.260 | 4,430 | 30 | 2 | 1 |
| BAT3 | 0.166 | 2,783 | 20 | 23 | 1 | 0.225 | 4,865 | 30 | 12 | 1 | 0.322 | 5,550 | 40 | 16 | 1 |
| BAT2 | 0.292 | 5,085 | 30 | 22 | 1 | 0.262 | 5,550 | 40 | 27 | 1 | 0.346 | 5,974 | 40 | 17 | 1 |
| CDK6 | 0.304 | 5,297 | 30 | 2 | 1 | 0.334 | 6,821 | 40 | 7 | 1 | 0.246 | 4,178 | 30 | 2 | 1 |
| RBBP8 | 0.699 | 12,138 | 70 | 1 | 1 | 0.327 | 6,700 | 40 | 10 | 1 | 0.681 | 11,889 | 70 | 6 | 1 |
| SF3B4 | 0.813 | 14,175 | 80 | 5 | 1 | 0.067 | 1,805 | 20 | 2 | 1 | 0.818 | 14,387 | 90 | 1 | 1 |
| SH3GL3 | 0.628 | 10,946 | 70 | 26 | 1 | 0.534 | 10,157 | 60 | 30 | 2 | 0.658 | 11,519 | 70 | 12 | 1 |
| DNM3 | 0.425 | 7,343 | 50 | 17 | 1 | 0.397 | 7,922 | 50 | 52 | 2 | 0.155 | 2,557 | 20 | 57 | 2 |
| DGKE | 0.475 | 8,251 | 50 | 31 | 2 | 0.156 | 3,585 | 30 | 48 | 2 | 0.365 | 6,324 | 40 | 41 | 2 |
| PEX1 | 0.604 | 10,518 | 60 | 31 | 2 | 0.923 | 16,318 | 99 | 53 | 2 | 0.913 | 16,157 | 99 | 51 | 2 |
| LYN | 0.667 | 11,576 | 70 | 47 | 2 | 0.004 | 265 | 2 | 51 | 2 | 0.793 | 13,943 | 80 | 54 | 2 |
| GNA12 | 0.248 | 4,289 | 30 | 45 | 2 | 0.090 | 2,272 | 20 | 60 | 3 | 0.163 | 2,711 | 20 | 27 | 1 |
| ATXN3 | 0.797 | 13,901 | 80 | 40 | 2 | 0.072 | 1,914 | 20 | 85 | 3 | 0.643 | 11,240 | 70 | 41 | 2 |
| DEF6 | 0.273 | 4,755 | 30 | 79 | 3 | 0.057 | 1,582 | 10 | 29 | 1 | 0.616 | 10,728 | 70 | 24 | 1 |
| BMP2 | 0.570 | 9,985 | 60 | 81 | 3 | 0.608 | 11,382 | 70 | 25 | 1 | 0.271 | 4,629 | 30 | 35 | 2 |
| RPS20 | 0.587 | 10,249 | 60 | 66 | 3 | 0.015 | 575 | 4 | 23 | 1 | 0.744 | 13,046 | 80 | 50 | 2 |
| SOCS2 | 0.523 | 9,159 | 60 | 73 | 3 | 0.012 | 509 | 3 | 64 | 3 | 0.343 | 5,928 | 40 | 81 | 3 |
| MOS | 0.583 | 10,181 | 60 | 68 | 3 | 0.016 | 617 | 4 | 115 | 4 | 0.564 | 9,743 | 60 | 92 | 4 |
| EFEMP1 | 0.291 | 5,056 | 30 | 93 | 4 | 0.000 | 4 | 1 | 104 | 4 | 0.822 | 14,466 | 90 | 217 | 10 |
| TRIP11 | 0.655 | 11,378 | 70 | 92 | 4 | 0.047 | 1,359 | 10 | 117 | 5 | 0.896 | 15,836 | 90 | 96 | 4 |
| HMGA2 | 0.418 | 7,241 | 50 | 143 | 5 | 0.011 | 494 | 3 | 396 | 20 | 0.118 | 1,937 | 20 | 257 | 10 |
| CPSF2 | 0.740 | 12,863 | 80 | 127 | 5 | 0.063 | 1,716 | 10 | 291 | 20 | 0.545 | 9,401 | 60 | 166 | 10 |
| GDF5 | 0.838 | 14,613 | 90 | 168 | 10 | 0.002 | 181 | 2 | 119 | 5 | 0.993 | 17,620 | 99 | 119 | 5 |
| BMP6 | 0.986 | 17,418 | 99 | 168 | 10 | 0.675 | 12,493 | 80 | 119 | 5 | 0.059 | 972 | 10 | 119 | 5 |
| HMGA1 | 0.996 | 17,639 | 99 | 222 | 10 | 0.000 | 19 | 1 | 357 | 20 | 0.337 | 5,814 | 40 | 167 | 10 |
| PXMP3 | 0.077 | 1,304 | 10 | 309 | 20 | 0.872 | 15,551 | 90 | 140 | 5 | 0.084 | 1,395 | 10 | 272 | 10 |
| NOG | 0.023 | 401 | 3 | 376 | 20 | 0.831 | 14,945 | 90 | 185 | 10 | 0.214 | 3,612 | 30 | 199 | 10 |
| PLAG1 | 0.461 | 7,988 | 50 | 389 | 20 | 0.000 | 2 | 1 | 198 | 10 | 0.961 | 17,038 | 99 | 386 | 20 |
| FBLN5 | 0.742 | 12,905 | 80 | 518 | 20 | 0.000 | 48 | 1 | 554 | 20 | 0.975 | 17,309 | 99 | 566 | 20 |
| CENTA2 | 0.937 | 16,414 | 99 | 306 | 20 | 0.352 | 7,145 | 50 | 419 | 20 | 0.933 | 16,528 | 99 | 305 | 20 |
| NCR3 | 0.489 | 8,493 | 50 | 632 | 30 | 0.559 | 10,551 | 70 | 1124 | 40 | 0.983 | 17,439 | 99 | 355 | 20 |
| TRIM25 | 0.405 | 7,006 | 40 | 1273 | 50 | 0.297 | 6,183 | 40 | 1050 | 40 | 0.584 | 10,131 | 60 | 1000 | 40 |
| TGS1 | 0.878 | 15,274 | 90 | 2689 | 99 | 0.608 | 11,377 | 70 | 2645 | 99 | 0.993 | 17,614 | 99 | 2298 | 90 |
