# Supplementary material for: A Network-Based Approach to Prioritize Results from Genome-Wide Association Studies
Source: PLoS One. 2011 Sep 6;6(9):e24220. doi: 10.1371/journal.pone.0024220 (PMC3168369; doi:10.1371/journal.pone.0024220)
Supplement: Table S2 — ‘Trait prioritized sub-networks’ for height. (DOC) [file pone.0024220.s006.doc]

**Table S2: ‘Trait prioritized sub-networks’ for height**

| **Bonferroni-corrected p-value** | |  | **David GO Set 1*** | **Genes in Set 1** | **Enrichment p-value** | **David GO Set 2*** | **Genes in Set 2** | **Enrichment p-value** |
| --- | --- | --- | --- | --- | --- | --- | --- | --- |
| **InCHIANTI** | **Korean** | **GAIN Controls** |
| 8.99E-18 | 1.62E-15 | 3.84E-13 | E | 76/129 | 5.40E-22 | N | 56/129 | 1.30E-11 |
| 1.91E-17 | 2.58E-14 | 1.36E-11 |  | 46/113 | 1.10E-24 | R | 66/113 | 3.30E-29 |
| 9.04E-22 | 1.72E-16 | 2.07E-19 |  | 104/119 | 2.20E-46 | Z | 47/119 | 6.20E-10 |
| 2.13E-15 | 4.33E-15 | 2.07E-16 |  | 28/95 | 7.70E-04 | Z | 24/95 | 1.90E-04 |
| 2.06E-13 | 1.28E-10 | 6.21E-11 |  | 100/172 | 6.90E-31 |  |  |  |
| 1.91E-11 | 5.34E-11 | 9.35E-12 | M | 37/96 | 2.00E-14 |  |  |  |
| 1.70E-12 | 1.53E-12 | 2.36E-12 |  | 53/100 | 2.00E-05 |  |  |  |
| 6.08E-19 | 1.12E-18 | 9.88E-23 | N | 102/167 | 3.60E-22 | E | 96/167 | 1.70E-21 |
| 7.14E-13 | 5.08E-12 | 4.68E-14 |  | 67/136 | 1.50E-11 | E | 65/136 | 1.90E-08 |
| 5.05E-17 | 1.72E-20 | 2.07E-10 |  | 56/130 | 1.50E-07 | E | 52/130 | 3.00E-07 |
| 5.43E-16 | 1.80E-12 | 1.74E-11 |  | 53/103 | 7.60E-15 | R | 65/103 | 4.10E-31 |
| 8.56E-19 | 1.82E-21 | 5.00E-20 |  | 43/121 | 2.40E-05 | Z | 30/121 | 8.90E-03 |
| 4.05E-16 | 9.04E-15 | 2.34E-16 |  | 41/94 | 6.30E-04 | Z | 24/94 | 8.90E-03 |
| 9.41E-15 | 3.55E-13 | 1.51E-14 |  | 41/97 | 2.00E-03 | Z | 25/97 | 7.70E-05 |
| 3.01E-15 | 5.27E-15 | 4.71E-15 |  | 54/123 | 1.10E-04 | Z | 31/123 | 1.40E-05 |
| 1.13E-15 | 5.03E-15 | 2.74E-16 |  | 40/94 | 9.80E-04 | Z | 24/94 | 8.40E-05 |
| 2.23E-15 | 1.46E-16 | 8.35E-17 |  | 30/98 | 6.10E-03 | Z | 27/98 | 5.70E-05 |
| 9.78E-15 | 2.04E-14 | 2.60E-15 |  | 40/94 | 2.10E-03 | Z | 24/94 | 1.50E-04 |
| 1.03E-14 | 2.99E-13 | 2.15E-15 |  | 32/121 | 1.60E-03 |  |  |  |
| 5.75E-21 | 1.43E-17 | 1.99E-20 |  | 47/140 | 4.70E-07 |  |  |  |
| 3.62E-13 | 1.75E-14 | 5.62E-15 |  | 50/165 | 3.40E-06 |  |  |  |
| 3.34E-19 | 4.91E-17 | 7.95E-22 |  | 40/134 | 9.10E-05 |  |  |  |
| 1.03E-15 | 1.38E-17 | 2.89E-17 |  | 55/125 | 1.60E-04 |  |  |  |
| 3.74E-19 | 9.14E-20 | 3.67E-19 |  | 62/159 | 6.30E-04 |  |  |  |
| 6.71E-16 | 1.06E-13 | 1.11E-11 |  | 55/136 | 2.80E-03 |  |  |  |
| 2.64E-12 | 1.16E-11 | 6.02E-12 | P | 53/161 | 2.10E-15 | S | 68/161 | 9.70E-20 |
| 1.79E-13 | 5.57E-13 | 1.29E-16 | R | 36/99 | 1.40E-33 |  |  |  |
| 1.67E-14 | 4.18E-12 | 1.49E-13 |  | 58/115 | 6.40E-52 |  |  |  |
| 2.19E-15 | 8.50E-11 | 1.37E-10 |  | 69/108 | 1.50E-23 |  |  |  |
| 1.21E-13 | 1.79E-13 | 2.60E-17 | S | 72/148 | 2.30E-05 | T | 60/148 | 3.50E-05 |
| 1.57E-13 | 2.26E-15 | 9.43E-16 | T | 92/141 | 1.60E-30 |  |  |  |
| 2.07E-10 | 2.69E-12 | 6.79E-12 | Z | 38/146 | 1.40E-04 |  |  |  |
| 5.79E-12 | 3.00E-11 | 1.21E-12 |  | 41/137 | 5.40E-05 |  |  |  |
| 8.23E-18 | 5.70E-19 | 1.00E-18 |  | 30/116 | 1.10E-04 |  |  |  |
|  |  |  |  |  |  |  |  |  |
| *E - Gene Expression; M - Protein metabolic process/protein modification process; N - Nucleic acid metabolism/Nucliec acid binding/DNA-Replication; P - Phosphate/phosphorus metabolic process; R - RNA processing/RNA binding/RNA metabolic process/RNA splicing/Transcription/Transcription Regulation; S - Signal transduction/Intracellular signaling/Cell communication; T - Transport/localization; Z - metal ion binding/zinc ion binding | | | | | | | | |
