# Supplementary material for: A Network-Based Approach to Prioritize Results from Genome-Wide Association Studies
Source: PLoS One. 2011 Sep 6;6(9):e24220. doi: 10.1371/journal.pone.0024220 (PMC3168369; doi:10.1371/journal.pone.0024220)
Supplement: Table S3 — Empirical p-values of height-prioritized sub-networks. (DOC) [file pone.0024220.s007.doc]

**Table S3: Empirical p-values of height-prioritized sub-networks**

| Sub-network | Occurances in 10,000 permutations | Empirical p-value | Suggestion to Users |
| --- | --- | --- | --- |
| 4640.out | 0 | <0.0001 | High priority |
| 4793.out | 0 | <0.0001 | High priority |
| 5362.out | 2 | 0.0002 | High priority |
| 2267.out | 21 | 0.0021 | High priority |
| 4085.out | 24 | 0.0024 | High priority |
| 3792.out | 30 | 0.0030 | High priority |
| 2982.out | 57 | 0.0057 | High priority |
| 4098.out | 61 | 0.0061 | High priority |
| 7084.out | 65 | 0.0065 | High priority |
| 3913.out | 71 | 0.0071 | High priority |
| 5375.out | 93 | 0.0093 | High priority |
| 7519.out | 123 | 0.0123 | High priority |
| 2421.out | 126 | 0.0126 | High priority |
| 6988.out | 336 | 0.0336 | High priority |
| 5808.out | 421 | 0.0421 | High priority |
| 6600.out | 435 | 0.0435 | High priority |
| 6796.out | 521 | 0.0521 | Lower priority |
| 6403.out | 686 | 0.0686 | Lower priority |
| 2579.out | 761 | 0.0761 | Lower priority |
| 5068.out | 1026 | 0.1026 | Lower priority |
| 727.out | 1096 | 0.1096 | Lower priority |
| 6931.out | 1097 | 0.1097 | Lower priority |
| 7632.out | 1122 | 0.1122 | Lower priority |
| 3993.out | 1422 | 0.1422 | Lower priority |
| 6328.out | 1912 | 0.1912 | Lower priority |
| 4596.out | 2579 | 0.2579 | Lower priority |
| 799.out | 4232 | 0.4232 | Lower priority |
| 5784.out | 4769 | 0.4769 | Lower priority |
| 5646.out | 5648 | 0.5648 | Ignore |
| 6377.out | 6579 | 0.6579 | Ignore |
| 6682.out | 7248 | 0.7248 | Ignore |
| 715.out | 7407 | 0.7407 | Ignore |
| 7637.out | 8108 | 0.8108 | Ignore |
| 6599.out | 8380 | 0.8380 | Ignore |
| 2778.out | 8497 | 0.8497 | Ignore |
| 2354.out | 8700 | 0.8700 | Ignore |
| 4053.out | 9295 | 0.9295 | Ignore |
| 6136.out | 9781 | 0.9781 | Ignore |
